# Beamforming of Joint Polarization-Space Matched Filtering for Conformal Array

**DOI:** 10.1155/2013/589675

**Published:** 2013-12-31

**Authors:** Lutao Liu, Yilin Jiang, Liangtian Wan, Zuoxi Tian

**Affiliations:** ^1^Department of Information and Communication Engineering, University of Harbin Engineering, Harbin 150001, China; ^2^Science and Technology on Underwater Test and Control Laboratory, Dalian 116013, China

## Abstract

Due to the polarization mismatch of the antenna, the received signal suffers from energy loss. The conventional beamforming algorithms could not be applied to the conformal array because of the varying curvature. In order to overcome the energy loss of the received signal, a novel joint polarization-space matched filtering algorithm for cylindrical conformal array is proposed. First, the snapshot data model of the conformal polarization sensitive array is analyzed. Second, the analytical expression of polarization sensitive array beamforming is derived. Linearly constrained minimum variance (LCMV) beamforming technique is facilitated for the cylindrical conformal array. Third, the idea of joint polarization-space matched filtering is presented, and the principle of joint polarization-space matched filtering is discussed in detail. Theoretical analysis and computer simulation results verify that the conformal polarization sensitive array is more robust than the ordinary conformal array. The proposed algorithm can improve the performance when signal and interference are too close. It can enhance the signal-to-noise ratio (SNR) by adjusting the polarization of the elements of the conformal array, which matches the polarization of the incident signal.

## 1. Introduction

Conformal array is defined as a set of sensors or elements mounted on the curved surface [1]. Comparing with the ordinary array, it has obvious advantages, for example, a large coverage of angle with good radiation pattern characteristics, reduction of aerodynamic drag, space savings, potential increase in available aperture, and reduction or elimination of random included preset errors [2]. It can be used in a variety of areas such as radar, sonar, biomedical imaging, and wireless communications [3].

The analysis of conformal array is difficult because the structure of the array is too complicated. Generally, the element pattern is defined in the local coordinate. Unlike the ordinary arrays containing elements of the same pattern, a conformal array can have elements of different patterns. The Euler rotation transformation [4–6] is adopted to transform the parameter from the global coordinate to local coordinate, which is very effective for the pattern transformation.

The multiple parameter estimation technique for array antenna has been widely applied in many fields. The estimation of signal parameters via rotational invariance techniques (ESPRIT) was used for two-dimensional angle and polarization estimation [7]. A novel algorithm to estimate the direction of arrival (DOA) and the polarization of a completely polarized polynomial-phase signal of an arbitrary degree with a single polarized vector-sensor was proposed in [8]. The algorithm can estimate DOA and polarization parameter without a priori knowledge of the polynomial-phase signal's coefficients and the signal's frequency spectrum. An algorithm for joint high-resolution DOA and polarization estimation using real-world arrays was proposed, which was suitable for correlated and coherent signals [9]. Cramer-Rao lower bound (CRB) for DOA and polarization estimation with arbitrary multiport antennas was derived in [10]. It can be used to compare different algorithms. The maximum likelihood (ML) and minimum-variance distortionless response (MVDR) estimators for DOA and polarization parameters were derived in a multipath environment [11]. The polarization sensitive array usually consists of multicomponent polarization sensitive antenna, which is arranged in the space under certain order. The advantage of the polarization sensitive array is that when the multiple polarization signals cannot be distinguished in the space domain, they can be distinguished in the polarization domain by adjusting the polarization information of the different signals.

At present, there are not many parameter estimation algorithms for conformal array. The multiple signal classification (MUSIC) algorithm and subarray technique were adopted in [12, 13], but the interpolation error always exists and the computational complexity is tremendous. A blind parameter estimation algorithm using ESPRIT was proposed for DOA estimation of conformal array [14]. The ESPRIT algorithm based on polarization sensitive array to estimate DOA and polarization parameter was proposed for conformal array [15]. The MVDR beamforming for cylindrical conformal array was investigated in [2]. A general and systematic method for pattern analysis of conformal arrays was presented using geometric algebra [16]. A beamforming approach for conformal array based on geometric algebra was proposed in [17]. However, the algorithms in [2, 16, 17] are only limited to space filtering. The joint polarization-space matched filtering for conformal array has not been researched extensively thus far.

The tradition matched filtering algorithm is based on spatial filtering, but the polarization information is not utilized. The matched filtering performance could be improved by exploiting the polarization information. The contribution of this paper is that a novel joint polarization-space matched filtering for cylindrical conformal array is proposed. The principle of the adaptive filtering is illustrated. The LCMV beamforming for conformal polarization sensitive array is analyzed. The signal-to-noise ratio (SNR) would increase by adjusting the polarization of the elements of the conformal array, which matches the polarization of the incident signal. In the future work, we will focus on the application of the proposed algorithm [18–21].

The organization of this paper is structured as follows.  Section 2 introduces the mathematical data model of the conformal array.  Section 3 contains the core contribution of this paper, which is the principle of joint polarization-space matched filtering based on LCMV beamforming. The analytical expression of polarization sensitive array beamforming is derived.  Section 4 presents the simulation result.  Section 5 summarizes our conclusions.

## 2. Mathematical Data Model

### 2.1. The Array Configuration

Unlike ordinary circular arrays, conformal array has the shadow effect attribute to the metal cylinder, which means that for an incident signal from a special angle, not all of the antenna elements can receive this signal. The sub-array divided technique proposed in [13] is adopted in this paper. Four subarrays are divided evenly, and each sub-array is in charge of azimuth coverage of 90°. The parameters estimation results of four sub-arrays are synthesized in order to estimate the incident signals parameters for the scope of the entire space.

**Figure 1:**
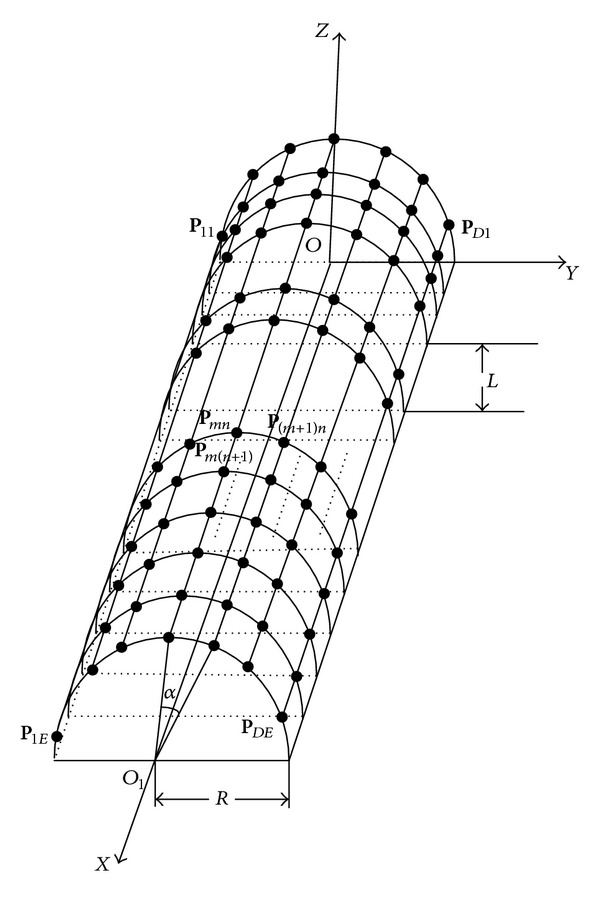
*D* × *E* cylindrical conformal array.

Consider a simple *D* × *E* cylindrical conformal array which is shown in Figure 1. There are *E* uniformly spaced rings on the surface of the cylinder perpendicular to the axis of symmetry. On each ring we have *D* orthogonal electric dipole pairs uniformly spaced. The radius of the cylinder is *R*. The distance between rings is *L* and the global coordinate is placed at the axis of the midpoint of the first ring. The angle between two consecutive antennas on a given ring is *α*. Where **P**_*mn*_ is the position vector of the (*m*, *n*)th element.

**Figure 2:**
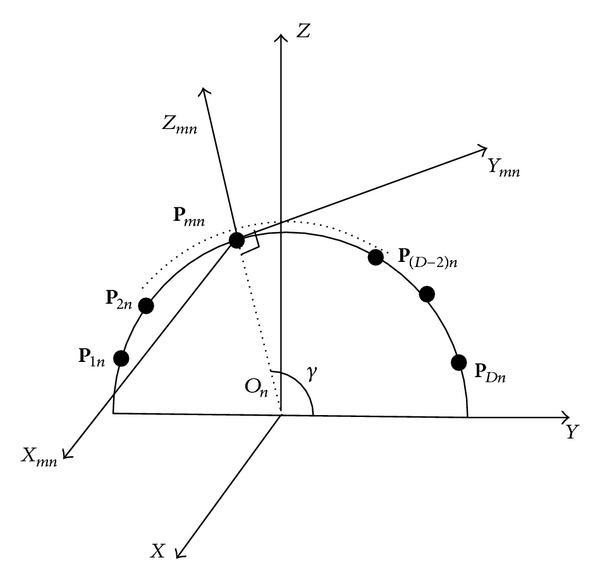
The local coordinate systems on the *n*th ring.

As shown in Figure 2, the (*m*, *n*)th element pattern is designed in element local Cartesian coordinate (*X*_*mn*_, *Y*_*mn*_, *Z*_*mn*_), and (*X*, *Y*, *Z*) represents the global Cartesian coordinate. The *X*_*mn*_-axis of element local Cartesian coordinate and the *X*-axis of the global Cartesian coordinate are parallel. *μ* is the angle between the *Z*_*mn*_-axis and *Y*-axis. The Cartesian coordinate of the unit vector with elevation *θ* and azimuth *φ* in the global coordinate can be expressed as

(1) $$\begin{array}{c} x=\sin\theta \cos\varphi ,\;\;y=\sin\theta \sin\varphi ,\;\;z=\cos\theta . \end{array}$$

With the array configuration and the coordinate design, the element pattern from the global coordinate to the local coordinate can be transformed as

(2) $$\begin{array}{c} x_{mn}=x,\\ y_{mn}=y\sin\left(\mu \right)-z\cos\left(\mu \right),\\ z_{mn}=y\cos\left(\mu \right)-z\sin\left(\mu \right). \end{array}$$

The Cartesian coordinate of the unit vector with elevation *θ* and azimuth *φ* in the local coordinates can be expressed as

(3) $$\begin{array}{c} \varphi_{mn}=\{\begin{array}{cc} \arctan\left(\frac{y_{mn}}{x_{mn}}\right), & x_{mn}>0,\\ \pi +\arctan\left(\frac{y_{mn}}{x_{mn}}\right), & x_{mn}<0, \end{array}\\ \theta_{mn}=\text{arccos}\left(z_{mn}\right). \end{array}$$

In order to avoid the condition shown in (3), the global coordinate is placed at the axis of the midpoint of the first ring. The transformation is simplified because **x**_*mn*_ = **x** > 0.

### 2.2. The Data Model Based on Polarization Sensitive Array

**Figure 3:**
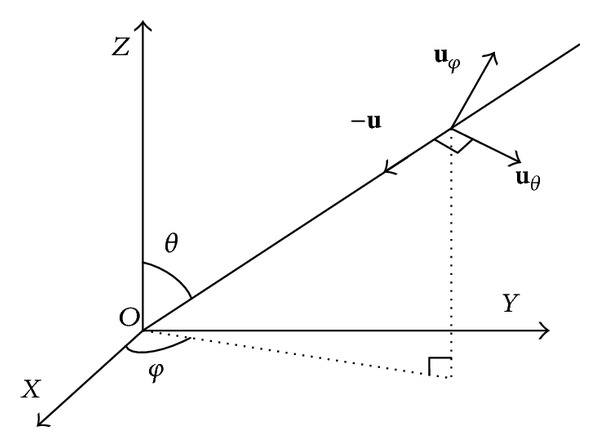
The direction vector **u** of the incident signal.

As shown in Figure 3, a completely polarized wave **u** is a narrowband far field source impinging on the array with elevation *θ* and azimuth *φ*. The definition and design of the element pattern are always in local coordinate; thus the patterns of each element in plane array are identical. However, for the conformal antenna array, the pattern of each element has different directions because of the varying curvature of conformal carrier. The modeling approach of steering vector based on plane array can no longer be used in this case. The new modeling approach of steering vector based on conformal antenna array is introduced as follows.

In global coordinate as shown in Figure 1, the steering vector of the cylindrical conformal array can be represented as

(4) a(θ,φ,γ,η)=[r1exp⁡(−j2πP1·uλ), r2exp⁡(−j2πP2·uλ),…, rDEexp⁡(−j2πPDE·uλ)]T,

(5) $$\begin{array}{c} u=\sin\left(\theta \right)\cos\left(\varphi \right)x+\sin\left(\theta \right)\sin\left(\varphi \right)y+\cos\left(\theta \right)z, \end{array}$$

where 0 ≤ *γ* < *π*/2 and −*π* ≤ *η* < *π* represent the auxiliary polarization angle and the polarization phase difference, respectively. **r**_*mn*_ is the response of the (*m*, *n*)th dipole to the unit strength incident signal. **P**_*mn*_ is the distance vector of the (*m*, *n*)th dipole which is in the global coordinate. *λ* is the wavelength of the incident signal.

The electric field vector in the global coordinates can be expressed as

(6) $$\begin{array}{c} E=E_{\theta }u_{\theta }+E_{\varphi }u_{\varphi },\\ u_{\theta }=\cos\left(\theta \right)\cos\left(\varphi \right)x+\cos\left(\theta \right)\sin\left(\varphi \right)y-\sin\left(\theta \right)z,\\ u_{\varphi }=-\sin\left(\varphi \right)x+\cos\left(\varphi \right)y, \end{array}$$

where **u**_*θ*_ and **u**_*φ*_ are the orthogonal unit component direction vectors of electric field. *E*_*θ*_ = sin*γ*exp⁡(*jη*) and *E*_*φ*_ = cos⁡*γ* are the component vectors by which the electric field vector is projected onto the **u**_*θ*_ and **u**_*φ*_, respectively.

In the local coordinate system as shown in Figure 2, the direction vector of the incident signal **u**′ and the electric field vector are given by

(7) u′=sin(θmn′)cos⁡(φmn′)x+sin(θmn′)sin(φmn′)y +cos⁡(θmn′)z,

(8) $$\begin{array}{c} E_{mn}^{\prime }=E_{\theta_{mn}^{\prime }}u_{\theta_{mn}^{\prime }}+E_{\varphi_{mn}^{\prime }}u_{\varphi_{mn}^{\prime }}, \end{array}$$

(9) uθ=cos⁡(θmn′)cos⁡(φmn′)x+cos⁡(θmn′)sin(φmn′)y −sin(θmn′)z,

where **u**_*θ*_*mn*_′_ and **u**_*φ*_*mn*_′_ are the orthogonal unit component direction vectors of electric field in local coordinate. *E*_*θ*_*mn*_′_ and *E*_*φ*_*mn*_′_ are the component vectors by which the electric field vector is projected onto **u**_*θ*_*mn*_′_ and **u**_*φ*_*mn*_′_, respectively. *θ*_*mn*_′ and *φ*_*mn*_′ are the signal's elevation and azimuth in the (*m*, *n*)th dipole's local coordinate, respectively.

Taking (2) into (9), then *E*_*θ*_*mn*_′_ and *E*_*φ*_*mn*_′_ are projected onto **u**_*θ*_ and **u**_*φ*_, respectively:

(10) $$\begin{array}{c} u_{\theta \theta_{mn}^{\prime }}=u_{\theta }\cdot u_{\theta_{mn}^{\prime }},\\ u_{\varphi \theta_{mn}^{\prime }}=u_{\varphi }\cdot u_{\theta_{mn}^{\prime }},\\ u_{\theta \varphi_{mn}^{\prime }}=u_{\theta }\cdot u_{\varphi_{mn}^{\prime }},\\ u_{\varphi \varphi_{mn}^{\prime }}=u_{\varphi }\cdot u_{\varphi_{mn}^{\prime }}. \end{array}$$

In local coordinate system, the electric field **E**_*mn*_′ vector is represented as

(11) Emn′=Eθmn′uθmn′+Eφmn′uφmn′=(Eθθmn′+Eφθmn′)uθmn′+(Eθφmn′+Eφφmn′)uφmn′=(Eθuθθmn′+Eφuφθmn′)uθmn′ +(Eθuθφmn′+Eφuφφmn′)uφmn′.

Two orthogonal electric field vectors *E*_*θ*_*mn*_′_ and *E*_*φ*_*mn*_′_ in the local coordinate can be expressed as follows using *E*_*θ*_ and *E*_*φ*_ which are defined in the global coordinate:

(12) $$\begin{array}{c} E_{\theta_{mn}^{\prime }}=E_{\theta }u_{\theta \theta_{mn}^{\prime }}+E_{\varphi }u_{\varphi \theta_{mn}^{\prime }},\\ E_{\varphi_{mn}^{\prime }}=E_{\theta }u_{\theta \varphi_{mn}^{\prime }}+E_{\varphi }u_{\varphi \varphi_{mn}^{\prime }}. \end{array}$$

Assume the direction pattern of the (*m*, *n*)th dipole is **g**_*mn*_(*θ*_*mn*_′, *φ*_*mn*_′). **g**_*θ*_*mn*__(*θ*_*mn*_′, *φ*_*mn*_′) and **g**_*φ*_*mn*__(*θ*_*mn*_′, *φ*_*mn*_′) are two component vectors by which **g**_*mn*_(*θ*_*mn*_′, *φ*_*mn*_′) is projected onto the **u**_*θ*_*mn*_′_ and **u**_*φ*_*mn*_′_, respectively. Then the dipole's response **p**_*mn*_ to unit electric vector is represented as

(13) pmn=gmn(θmn′,φmn′)Emn′=[gθmn(θmn′,φmn′)uθmn′  +gφmn(θmn′,φmn′)uφmn′] ×(Eθmn′uθmn′+Eφmn′uφmn′)=gθmn(θmn′,φmn′)Eθmn′uθmn′ +gφmn(θmn′,φmn′)Eφmn′uφmn′.

The polarized vector received by the dipole is represented as

(14) rmn=[uxmn′uymn′]=[gθmn(θmn′,φmn′)cos⁡(θmn′)cos⁡(φmn′)Eθmn′−gφmn(θmn′,φmn′)sin(φmn′)Eφmn′gθmn(θmn′,φmn′)cos⁡(θmn′)sin(φmn′)Eθmn′+gφmn(θmn′,φmn′)cos⁡(φmn′)Eφmn′]=[cos⁡(θmn′)cos⁡(φmn′)−sin(φmn′)cos⁡(θmn′)sin(φmn′)cos⁡(φmn′)]×([gθmn(θmn′,φmn′)gθmn(θmn′,φmn′)]⊗[Eθmn′Eφmn′]),

(15) $$\begin{array}{c} E_{mn}^{\prime }=\left[\begin{array}{c} E_{\theta_{mn}^{\prime }}\\ E_{\varphi_{mn}^{\prime }} \end{array}\right]=\left[\begin{array}{cc} u_{\theta \theta_{mn}^{\prime }} & u_{\varphi \theta_{mn}^{\prime }}\\ u_{\theta \varphi_{mn}^{\prime }} & u_{\varphi \varphi_{mn}^{\prime }} \end{array}\right]\left[\begin{array}{c} E_{\theta }\\ E_{\varphi } \end{array}\right]=U_{mn}\left[\begin{array}{c} \sin\gamma \exp\left(j\eta \right)\\ \cos\gamma \end{array}\right], \end{array}$$

where ⊗ represents the Kronecker product.

**Definition 1**

For two matrices **B** ∈ *𝒞* ^*B*×*L*^ and **D** ∈ *𝒞* ^*D*×*L*^, which possess the identical number of columns, the KR product of the two matrices is given by

(16) $$\begin{array}{c} B⊙D=\left[b_{1}⊗d_{1},b_{2}⊗d_{2},\ldots ,b_{L}⊗d_{L}\right]\in {𝒞}^{BD\times L}, \end{array}$$

where ⊗ stands for the Kronecker product.

**Definition 2**

For two vectors **b** ∈ *𝒞* ^*B*^ and **d** ∈ *𝒞* ^*D*^, the Kronecker product of these two matrices is given by

(17) $$\begin{array}{c} b⊗d=\left[\begin{array}{c} b_{1}d\\ b_{2}d\\ \vdots \\ b_{B}d \end{array}\right]=\mathrm{vec}\left(db^{T}\right). \end{array}$$

Under the condition of *Q* signals impinging on the array, the manifold matrix is expressed as

(18) A=[a1(θ1,φ1,γ1,η1)a2(θ2,φ2,γ2,η2)⋯aQ(θQ,φQ,γQ,ηQ)].

The data model is established as

(19) $$\begin{array}{c} X\left(t\right)=AS\left(t\right)+N\left(t\right), \end{array}$$

(20) $$\begin{array}{c} S\left(t\right)={\left[\begin{array}{cccc} s_{1}\left(t\right) & s_{2}\left(t\right) & \cdots & s_{Q}\left(t\right) \end{array}\right]}^{T}, \end{array}$$

where **N**(*t*) is the noise matrix which is assumed to be a Gaussian process with zero mean and *σ* ^2^ variance and **S**(*t*) is the *Q* × 1 signal matrix.

The covariance matrix of **X**(*t*) is represented as

(21) $$\begin{array}{c} R_{X}=E\left[X\left(t\right)X^{H}\left(t\right)\right]=AE\left[S\left(t\right)S{\left(t\right)}^{H}\right]A^{H}+\sigma_{n}^{2}I, \end{array}$$

where *σ* ^2^ denotes the noise power and *E*[**S**(*t*)**S**(*t*)^*H*^] is the signal power.

In order to illuminate the pattern transformation, the cylinder is adopted as an example. The corresponding Euler rotation angles are

(22) $$\begin{array}{c} D=-\Theta ,\;\;E=-\frac{\pi }{2},\;\;F=0, \end{array}$$

respectively. *D* represents the rotation angle based on the right-hand rule in the first rotation and the *Z*-axis is the rotation axis; *E* represents the rotation angle in the second rotation and the *Y*-axis is the rotation axis; *F* represents the rotation angle in the third rotation and the *X*-axis is the rotation axis. Θ represents the angle between the position vector of the element and the positive direction of *X*-axis.

The unit vector of the *q*th incident signal (*θ*_*q*_, *φ*_*q*_) in the global coordinate can be expressed as

(23) $$\begin{array}{c} x=\sin\left(\theta_{q}\right)\cos\left(\varphi_{q}\right),\;\;y=\sin\left(\theta_{q}\right)\sin\left(\varphi_{q}\right),\\ z=\cos\left(\theta_{q}\right). \end{array}$$

Based on the Euler rotation matrix, the unit vector in global coordinate can be transformed into the local coordinate of the *m*th element:

(24) $$\begin{array}{c} {\left[\begin{array}{ccc} x_{m} & y_{m} & z_{m} \end{array}\right]}^{T}=R\left(D_{m},E_{m},F_{m}\right){\left[\begin{array}{ccc} x & y & z \end{array}\right]}^{T}, \end{array}$$

where

(25) R(Dm,Em,Fm)=[cos⁡FsinF0−sinFcos⁡F0001][cos⁡E0−sinE010sinE0cos⁡E] ×[cos⁡DsinD0−sinDcos⁡D0001].

Then the elevation *θ* and azimuth *φ* of the *k*th incident signal in the local coordinate of the *m*th element could be represented as

(26) $$\begin{array}{c} \varphi_{mq}=\arctan\left(\frac{y_{m}}{x_{m}}\right),\;\;\theta_{mq}=\text{arccos}\left(z_{m}\right). \end{array}$$

The element pattern transformation from the global coordinate to the local coordinate is completed at this time.

## 3. The Principle of Joint Beamforming

The polarized and spaced information of the target is assumed to be known. In order to maximize the power of the received signal, the weight vector should match the received signal on both polarized domain and spaced domain. By adjusting the weight vector value, it could match the received signal completely on both polarized domain and spaced domain, and the power of the received signal is maximized, which is called polarization-space beamforming (PSB).

Substituting (14) into (4), the steering vector is represented as

(27) wPSB=a(θ,φ,γ,η)=rexp⁡(−j2πP·uλ)=ap(θ,φ,γ,η)⊗as(θ,φ)=aps(θ,φ)app(γ,η)⊗as(θ,φ).

It can be seen from (20) that the polarization weight vector includes the polarization vector and spaced vector. However, the ordinary weight vector merely contains the spaced vector. Consider the condition that the polarization of the received signal is orthogonal to the polarization of the antenna; the polarization array has much advantage over ordinary array, because the polarization statue of the ordinary array is stationary, but the polarization statue of the polarization array can be changed flexibly. When the polarization of the polarization array and the received signal are identical, the polarization array can still possess a good performance. Based on the analysis above, it can be known that the polarization array has more robustness than the ordinary array.

In order to simplify the analysis, (17) is written in another form which contains desired signal, interference, and noise components, respectively:

(28) X(t)=a0s0(t)+∑i=1Jaisi(t)+N(t)=a0s0(t)+aJSJ(t)+N(t),

where *s*_0_(*t*) is the desired signal and *s*_1_(*t*), *s*_2_(*t*),…, *s*_*J*_(*t*) are interferences which are independent of both desired signal and noise.

Taking (21) into (19), the covariance matrix **R**_**X**_ can be written in another form:

(29) RX=E[X(t)XH(t)]=PSa0a0H+∑i=1JPiaiaiH+σn2I,

where *P*_*S*_ is the signal power and *P*_*i*_ is the *i*th interference noise. Let **P**_*in*_ = ∑_*i*=1_^*J*^ *P*_*i*_ **a**_*i*_ **a**_*i*_^*H*^ + *σ*_*n*_^2^ **I**; (23) can be obtained as:

(30) E[y(t)yH(t)]=wPSBHRXw=PSwPSBHa0a0Hw+wPSBHPinw.

The signal-to-interference-plus-noise ratio (SINR) can be expressed as (24) using (23):

(31) $$\begin{array}{c} {\text{SINR}}_{\text{PSB}}=\frac{P_{S}w_{\text{PSB}}^{H}a_{0}a_{0}^{H}w}{w_{\text{PSB}}^{H}P_{in}w}. \end{array}$$

### 3.1. No Interferences

The output of the polarization-spaced matched filtering under no interferences is expressed as

(32) $$\begin{array}{c} y\left(t\right)=w_{\text{PSB}}^{H}X\left(t\right)=a_{0}^{H}a_{0}s_{0}\left(t\right)+a_{0}^{H}N\left(t\right). \end{array}$$

The signal power that the antenna received is denoted by *σ*_*s*_^2^, so the desired signal power based on the array output is

(33) PS=E[|a0Ha0s0(t)|2]=(DE)2σs2apH(θ,φ,γ,η)ap(θ,φ,γ,η)=(DE)2σs2(aps(θ,φ)app(γ,η))Haps(θ,φ)app(γ,η).

The noise power based on the array output is

(34) $$\begin{array}{c} P_{n}=a_{0}^{H}N\left(t\right)N^{H}\left(t\right)a_{0}=DE\sigma_{n}^{2}. \end{array}$$

The signal-to-noise ratio (SNR) after the joint matched filtering is

(35) SNRPSB=DEσs2σn2(aps(θ,φ)app(γ,η))Haps(θ,φ)app(γ,η)=DE·SNRdiple,

where SNR_diple_ is the single dipole's SNR, which is determined by two parts: one is its own SNR of the signal *σ*_*s*_^2^/*σ*_*n*_^2^ and the other is the polarization vector **a**_*p*_(*θ*, *φ*, *γ*, *η*).

### 3.2. One Interference

Under the condition that one interference exists, linear constraints have been widely used in the adaptive beamforming. The weight vector of the LCMV beamforming is solved by minimizing the output power under a series of linear constraints **C** ^*H*^ **w** = **f**, where **C** is a *N* × *m* constraint matrix, **f** is *m* × 1 constraint value vector, and *N* is the number of elements. The optimization problem of LCMV beamforming is expressed as follows:

(36) $$\begin{array}{c} \underset{w}{\min}\;w^{H}R_{X}w\\ \text{s}.\text{t}.\;C^{H}w=f. \end{array}$$

This optimization problem can be solved by the Lagrange multiplier method. The optimal weight vector is represented as follows:

(37) $$\begin{array}{c} w_{c}=R_{X}^{-1}C{\left(C^{H}R_{X}^{-1}C\right)}^{-1}f, \end{array}$$

where **C** and **f** are the constraint matrix and response vector, respectively. In this paper, the unit gain constraint **a**_*e*_^*H*^ **w**_*c*_ = 1 is adopted, and **a**_*e*_ is the explored signal vector. Under ideal condition, **a**_*e*_ = **a**_0_. The polarization information and spaced information of the desired signal is already known, in which the unit gain constraint is to be formed. Then (29) can be transformed as

(38) $$\begin{array}{c} w_{c}=R_{X}^{-1}a_{e}{\left(a_{e}^{H}R_{X}^{-1}a_{e}\right)}^{-1}f. \end{array}$$

Let *η* = **a**_*e*_(**a**_*e*_^*H*^ **R**_*X*_^−1^ **a**_*e*_)^−1^; (31) is written as

(39) $$\begin{array}{c} w_{c}=\eta R_{X}^{-1}a_{e}. \end{array}$$

Based on (16), **R**_**X**_^−1^ can be obtained by using matrix inversion theorem:

(40) $$\begin{array}{c} R_{X}^{-1}={\left(P_{in}+P_{S}a_{0}a_{0}^{H}\right)}^{-1}=P_{in}^{-1}-\frac{P_{S}P_{in}^{-1}a_{0}a_{0}^{H}P_{in}^{-1}}{1+P_{S}a_{0}^{H}P_{in}^{-1}a_{0}}. \end{array}$$

Taking (33) into (32), then let **a**_*e*_ = **a**_0_; **w**_*c*_ is represented in another simple form as

(41) $$\begin{array}{c} w_{c}=\frac{\eta P_{in}^{-1}a_{0}}{1+P_{S}a_{0}^{H}P_{in}^{-1}a_{0}}. \end{array}$$

The SINR of the polarization-spaced LCMV beamforming is expressed as follows using (34):

(42) $$\begin{array}{c} \text{SINR}=\frac{P_{S}w_{c}^{H}a_{0}a_{0}^{H}w_{c}}{w_{c}^{H}P_{in}w_{c}}=P_{S}a_{0}^{H}P_{in}^{-1}a_{0}. \end{array}$$

When one interference merely exists, **P**_*in*_ = *P*_1_ **a**_1_ **a**_1_^*H*^ + *σ*_*n*_^2^ **I**,

(43) $$\begin{array}{c} P_{in}^{-1}=\frac{1}{\sigma_{n}^{2}}\left(I-\frac{P_{1}a_{1}a_{1}^{H}}{\sigma_{n}^{2}+P_{1}{||a_{1}||}^{2}}\right). \end{array}$$

Taking (36) into (35), SINR of one interference is acquired.

(44) $$\begin{array}{c} {\text{SINR}}_{\text{one}}=\frac{P_{S}{||a_{0}||}^{2}}{\sigma_{n}^{2}+P_{1}{||a_{1}||}^{2}}+\frac{P_{S}P_{1}{||a_{0}||}^{2}{||a_{1}||}^{2}}{\sigma_{n}^{2}\left(\sigma_{n}^{2}+P_{1}{||a_{1}||}^{2}\right)}\left(1-{\left|\tau \right|}^{2}\right), \end{array}$$

(45) $$\begin{array}{c} {\left|\tau \right|}^{2}=\frac{a_{0}^{H}a_{1}a_{1}^{H}a_{0}}{{||a_{0}||}^{2}{||a_{1}||}^{2}}. \end{array}$$

The correlation between desired signal and interference is reflected by |*τ*|:

(46) $$\begin{array}{c} a_{0}^{H}a_{1}={\left(a_{p0}⊗a_{s0}\right)}^{H}\left(a_{p1}⊗a_{s1}\right)=\left(a_{p0}^{H}a_{p1}\right)\left(a_{s0}^{H}a_{s1}\right),\\ a_{1}^{H}a_{0}={\left(a_{p1}⊗a_{s1}\right)}^{H}\left(a_{p0}⊗a_{s0}\right)=\left(a_{p1}^{H}a_{p0}\right)\left(a_{s1}^{H}a_{s0}\right),\\ {||a_{0}||}^{2}={||a_{p0}||}^{2}\cdot {||a_{s0}||}^{2},\\ {||a_{1}||}^{2}={||a_{p1}||}^{2}\cdot {||a_{s1}||}^{2}. \end{array}$$

|*τ*| could be written in another form as

(47) $$\begin{array}{c} \left|\tau \right|=\frac{\left(a_{p0}^{H}a_{p1}\right)\left(a_{p1}^{H}a_{p0}\right)}{{||a_{p0}||}^{2}{||a_{p1}||}^{2}}\frac{\left(a_{s0}^{H}a_{s1}\right)\left(a_{s1}^{H}a_{s0}\right)}{{||a_{s0}||}^{2}{||a_{s1}||}^{2}}=M_{p}M_{s}, \end{array}$$

where *M*_*p*_ is polarization match coefficient and *M*_*s*_ is spaced match coefficient. Obviously, 0 < *M*_*p*_ < 1, 0 < *M*_*s*_ < 1.

### 3.3. Multiinterferences

Consider multiinterferences impinging on the array; it is too complicated to calculate the inversion of the covariance matrix **P**_*in*_. Instead, an iterative method is used here:

(48) (Pin(k))−1=(Pin(k−1)+Pi(k)akakH)−1=(Pin(k−1))−1−Pi(k)(Pin(k−1))−1akakH(Pin(k−1))−11+Pi(k)akH(Pin(k−1))−1ak.

According to the recursive relation of **P**_*in*_ as shown in (43), the recursive relation of SINR is represented as

(49) SINR(k)=PSa0HPin−1a0=PSa0H((Pin(k−1))−1−Pi(k)(Pin(k−1))−1akakH(Pin(k−1))−11+Pi(k)akH(Pin(k−1))−1ak)a0=SINR(k−1)−PSPi(k)|a0H(Pin(k−1))−1ak|21+Pi(k)akH(Pin(k−1))−1ak.

It can be seen from (44) that SINR decreases as the number of interferences increases. When multiinterferences exist, SINR not only depends on the correlation between desired signal and interference, but also depends on the correlation among the interferences.

## 4. Simulation Results

Three simulation texts were performed corresponding to the conditions that no interferences exist, one interference exists, or multiinterferences exist, respectively. The element pattern used in simulation is the lowest order circular patch model:

(50) gθ(θ,φ)={J2(πdλsinθ)−J0(πdλsinθ)} ×(cos⁡φ−jsinφ), 0≤θ≤π2,

(51) gφ(θ,φ)={J2(πdλsinθ)+J0(πdλsinθ)} ×cos⁡θ(sinφ−jcos⁡φ), 0≤θ≤π2,

(52) $$\begin{array}{c} g_{\theta }\left(\theta ,\varphi \right)=g_{\varphi }\left(\theta ,\varphi \right)=0,\;\text{otherwise}, \end{array}$$

where *J*_0_ and *J*_2_ are the zeroth- and second-order Bessel functions of the first kind. The element pattern transformation is completed with the method proposed above.

**Simulation**

**Figure 4:**
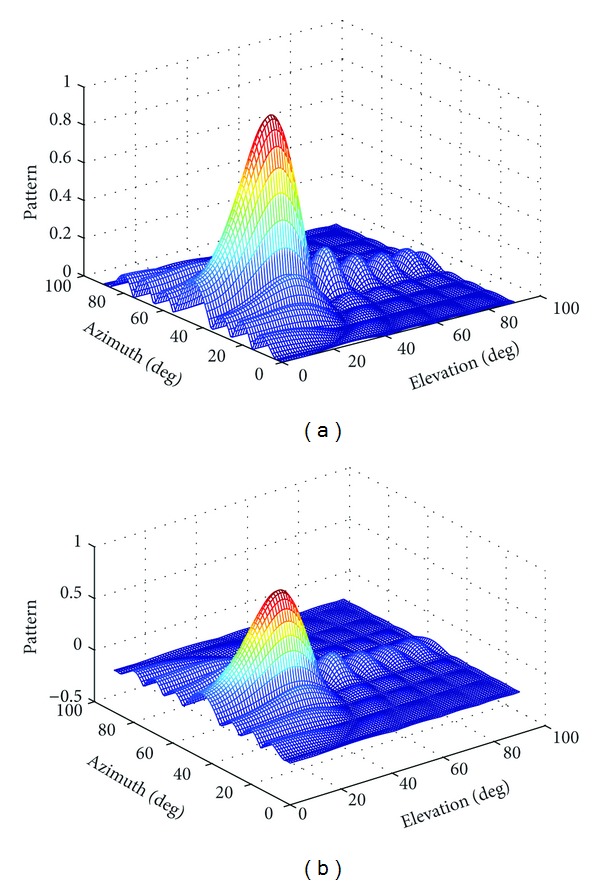
The array pattern. (a) The pattern of polarization array. (b) The pattern of ordinary array.

To demonstrate the effectiveness of the proposed joint polarization-space matched filtering algorithm, a 5 × 5 cylindrical conformal array is used in the simulation. The normalized patterns of polarization array and ordinary array mounted on conformal carrier are depicted in Figures 4(a) and 4(b), respectively. The frequency of the incident signal is *f* = 2 GHz, *λ* = *c*/*f*, *c* = 3 × 10^8^ m/s is light velocity; *α* = 5°, and the distance between rings is *L* = *λ*/2. The radius of the cylinder is *R* = *L*/*α*. The polarization parameter and DOA are (*γ*_0_, *η*_0_) = (45°, 270°) and (*θ*_0_, *φ*_0_) = (30°, 45°), respectively.  Figure 4(a) shows the pattern of the polarization array; the maximum gain happens at the direction of the incident signal.  Figure 4(b) shows the pattern of ordinary array when the polarization parameter is (*γ*_0_, *η*_0_) = (45°, 315°). The polarization weights could not be adjusted in ordinary array. The polarization mismatch exists between the element and incident signal, and the pattern gain is lower than that of the polarization array. However, the shapes of the polarization array and ordinary array are similar, which is consistent with the theoretical analysis.

**Simulation**

**Figure 5:**
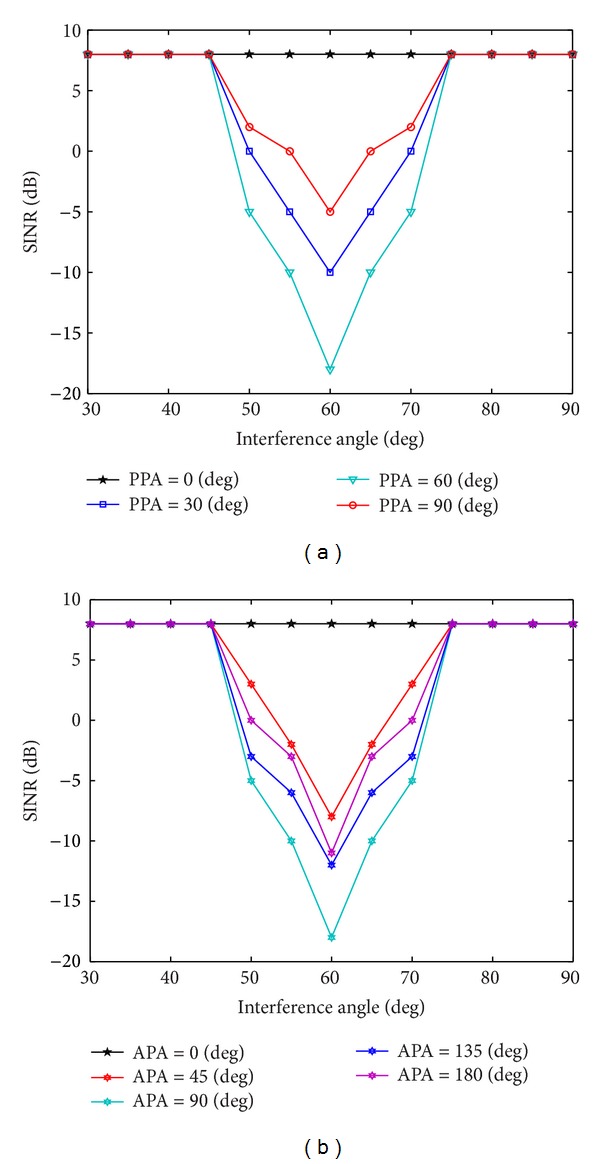
The SINR under different polarization parameters. (a) The SINR under different polarization phase angles. (b) The SINR under different auxiliary polarization angles.

Only the uniform linear array (ULA) which is perpendicular to the “ring” is considered for array matched filtering. The elevation and azimuth of the desired signal is (*θ*_1_, *φ*_1_) = (60°, 0°), and the polarization parameter is (*γ*_1_, *η*_1_) = (90°, 60°), SNR = 0 dB, and INR = 20 dB. The azimuth range of interference signal is zero. The elevation range of interference signal is 30°–90°. Other simulation conditions are the same as those in Simulation 1. As shown in Figure 5(a), the polarization phase angle (it is short for PPA in Figure 5(a)) of the interference signal is *η*_2_ = 60°, and the elevation of the interference signal is *γ*_2_ ∈ (0°, 90°); the step-length is 30°. As shown in Figure 5(b), the auxiliary polarization angle (it is short for APA in Figure 5(b)) of the interference signal is *γ*_2_ = 90°, and the elevation of the interference signal is *η*_2_ ∈ (0°, 180°); the step-length is 45°. In Figure 5, we can see that when the desired signal and interference signal are close (|*θ*_2_ − *θ*_1_| < 10°), the polarization information plays an important role in improving the performance of filtering. The larger the difference between desired signal and interference signal, the better performance of filtering. When the desired signal and interference signal are relatively far apart (|*θ*_2_ − *θ*_1_| > 15°), the space information plays an important role in improving the performance of filtering; the function of polarization information is not obvious.

**Simulation**

The following LCMV beamforming experiment demonstrates that the joint polarization-space matched filtering technique can be applied to beamforming for conformal array effectively. The whole space is all included. The noise variance is assumed to be *σ* ^2^ = 1. The SNR = −10 dB, and INR = 20 dB. There are six incident signals impinging on the conformal array:

1. the desired signal from (*φ*_*s*_, *θ*_*s*_) = (180°, 85°),
2. the first interference signal from (*φ*_*i*1_, *θ*_*i*1_) = (80°, 15°),
3. the second interference signal from (*φ*_*i*2_, *θ*_*i*2_) = (100°, 45°),
4. the third interference signal from (*φ*_*i*3_, *θ*_*i*3_) = (130°, 60°),
5. the fourth interference signal from (*φ*_*i*4_, *θ*_*i*4_) = (200°, 50°),
6. the fifth interference signal from (*φ*_*i*5_, *θ*_*i*5_) = (250°, 70°).

**Figure 6:**
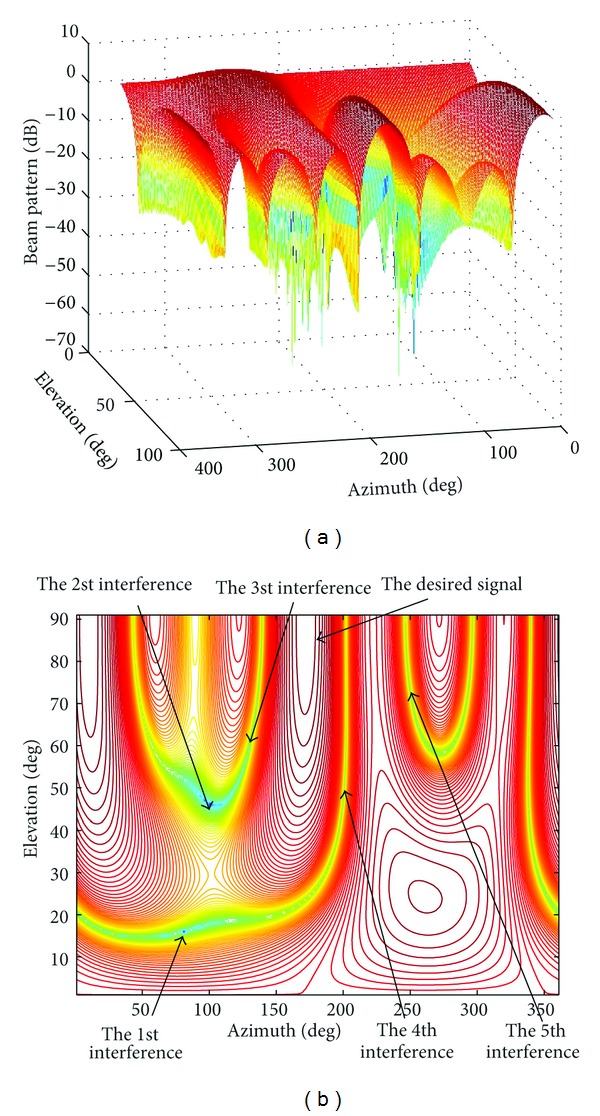
The LCMV beamforming. (a) Beamforming 3D pattern. (b) Contour plot.

Other simulation conditions are the same as the ones in Simulation 1. As shown in Figure 6, the LCMV beamforming approach sets five null points on the five interferences. The main beam is pointed at the desired signal. The proposed algorithm is very effective for beamforming of conformal array.

## 5. Conclusion

In this paper, a novel joint polarization-space matched filtering approach for conformal array is proposed. The analytical expression of polarization sensitive array beamforming is derived, which is equivalent to the matched coefficient multiplying the beamforming of the ordinary array. The polarization sensitive array has a higher gain by adjusting the polarization of the elements. Simulation results demonstrate that the joint polarization-space matched filtering can improve the filtering performance of the conformal array effectively.
